# Proteomic characterization of fresh spermatozoa and supernatant after cryopreservation in relation to freezability of carp (*Cyprinus carpio* L) semen

**DOI:** 10.1371/journal.pone.0192972

**Published:** 2018-03-22

**Authors:** Mariola A. Dietrich, Andrzej Ciereszko

**Affiliations:** Department of Gametes and Embryo Biology, Institute of Animal Reproduction and Food Research, Polish Academy of Sciences, Tuwima, Olsztyn, Poland; Chung-Ang University, REPUBLIC OF KOREA

## Abstract

Our recent studies suggested that the freezability of carp semen is related to seminal plasma protein profiles. Here, we aimed to compare the spermatozoa proteomes of good (GF) and poor (PF) freezability semen of carp. To achieve this, we used two-dimensional difference in gel electrophoresis followed by MALDI-TOF/TOF mass spectrometry. The semen was classified as GF or PF based on sperm motility after freeze/thawing. We identified proteins enriched in spermatozoa of GF (22 proteins) and PF (18 proteins) semen. We also identified 12 proteins enriched in the supernatant after cryopreservation of PF semen. Good freezability is related to high concentrations of proteins involved in the maintenance of flagella structure, membrane fluidity, efficient control of Ca^2+^ and sperm motility, energy production, and antioxidative protection, which likely reflects the full maturation status of spermatozoa of GF semen. On the other hand poor freezability seems to be related to the presence of proteins identified as released in high quantities from cryopreserved sperm of PF. Thus, the identified proteins might be useful bioindicators of freezing resilience and could be used to screen carp males before cryopreservation, thus improve long-term sperm preservation in carp. Data are available via ProteomeXchange with identifier PXD008187.

## Introduction

Cryopreservation has been extensively used in assisted reproductive technology, agriculture, and conservation programs for endangered species. However, in fish breeding, this method is not yet implemented on a commercial level. Cryopreservation is a damaging process that induces oxidative and osmotic stresses, which alter lipid and protein composition, decrease motility and viability, cause damage to mitochondria and sperm tails, and increase sperm DNA fragmentation leading to a decrease in vertebrate sperm quality after cryopreservation [1–7]. For those reasons, cryopreservation protocols have to be carefully optimized in order to minimalize the above-mentioned damages. Several effective protocols for the cryopreservation of carp semen have been established [8–11]. However, these protocols do not produce satisfactory results for some individuals because of differences in samples ability to withstand the freezing-thawing process [12]. Therefore, the identification of markers for predicting carp semen cryopreservation outcomes is a prerequisite for improving sperm cryopreservation protocols.

The quality parameters of fresh semen (e.g., motility, viability and sperm concentration) have been used as predictive tools of sperm cryopreservation potential in fish [5, 6, 9, 13, 14]. However, the usefulness of such bioindicators varies across fish species and between individuals. For this reason, there is a need to identify molecular biomarkers of semen quality. In mammals, such freezability differences have been related to protein composition of seminal plasma and spermatozoa [15–19]. Some specific sperm protein markers of good and poor semen freezability have been identified in mammals [20, 21] Higher levels of heat shock protein 90, acrosin binding protein and voltage-dependent anion channel 2 and lower levels of triosephosphate isomerase are correlated with GF in boar sperm [22–26]. High levels of enolase and glucose-6-phosphate isomerase have been shown to be markers of GF in human semen [27]. A recent comparative analysis of the bull sperm proteome revealed that high freezability is related to higher levels of proteins associated with stabilization of acrosome structure, sperm membrane stabilization and sperm energy metabolism [28]. Furthermore proteomic studies in humans and bulls revealed that sperm proteins that are changed by cryopreservation could also serve as markers of resilience to long-term cryopreservation [29, 30]. Yoon et al. [31] observed epididymal sperm proteome dynamics and possible protein markers of cryo-stress during cryopreservation. They found that nine proteins were differentially expressed before and after cryopreservation (levels of two proteins decreased and levels of seven increased). As transcription, translation and protein synthesis generally do not occur in mature spermatozoa the observed increase in protein abundance after cryopreservation may be a result of post-translational modification.

The percentage of motile sperm after freezing and thawing is commonly used as an indicator of sperm quality in both mammals and fish. It has been used in the dairy industry to evaluate the success of cryopreservation and as a discriminating factor in proteomic research on the freezability of mammalian semen [17–19, 32]. Recently Horokhorvatskyi et al. [12] used post-thaw sperm motility to classify the cryoresistance of carp semen. In fish CASA assessment of the percentage of motile sperm in fresh and cryopreserved semen samples is positively correlated with fertilizing ability and it can be used as a proxy for fertilization capacity in multiple fish species, including carp [14, 33–35]. At present it is unknown whether other sperm characteristics can be used as indicators of semen quality. An earlier study found that sperm viability in fish was a less sensitive indicator of heavy metal toxicity and fertilizing ability than sperm motility [36].

Semen consist in spermatozoa and surrounding fluid called seminal plasma. Our recent studies have suggested that carp semen freezability is related to seminal plasma protein profiles, even when the tested semen are characterized by similar initial sperm quality parameters [37]. We have indicated in seminal plasma potential protein biomarkers of freezability related to sperm membrane integrity and antioxidative protection. However, to the best of our knowledge, there is no information concerning the relationship between proteome of carp spermatozoa and freezability. Such study should provide additional novel biomarkers of semen freezability in carp spermatozoa. This knowledge is a prerequisite for better understanding of the mechanism of cryoinjuries to particular structures and functions of spermatozoa.

The aim of the present study was to compare the proteome of fresh (before cryopreservation) spermatozoa of GF and PF carp semen using 2D-DIGE technique and to identify differentially abundant proteins. Moreover we compared the supernatants of GF and PF semen after cryopreservation and identified proteins leaked in greater quantities from the spermatozoa of PF than GF. The identified sperm proteins can serve as potential biomarkers of freezability. We believe that based on the results we obtained ELISA tests could be developed to screen male carp for good sperm freezability and to improve preservation techniques in carp.

## Material and methods

### Fish origin and broodstock management

The milt of the common carp (*Cyprinus carpio* L) was obtained from fish maintained at the Institute of Ichthyobiology and Aquaculture of the Polish Academy of Sciences in Gołysz, Poland. Males were randomly selected from a larger population of spawners from pond in the middle of the spawning season (July 2nd). The group of 5- to 7-year- old males (n = 20) was placed in a 2.5 m3 tank filled with aerated water at 19–20°C and maintained under stable conditions for a two-day adaptation period. Twenty-four hours before the collection of carp semen, the males were injected intradorsally with Ovopel (one pellet containing 18–20 μg of a GnRH analogue and 8–10 mg of metoclopramide per kg of fish body weight; Interfish Ltd., Hungary). The milt was obtained by gentle abdominal massage, taking care not to pollute the sample with blood, feces or urine.

Approval from the Committee on the Ethics of Animal Experiments in Olsztyn, Poland (no. 93/2011) was obtained before starting the experiments.

### Collection of good and poor freezability semen

This study is a continuation of our recently published report on differences in seminal plasma protein profile in carp semen with good freezability (GF) and poor freezability (PF) [37]. The same GF (n = 6) and PF (n = 6) semen samples were used for the analyses of seminal plasma [37] and spermatozoa (this study).

To distinguish semen samples between two groups, semen was cryopreserved using the freezing method described by Kopeika [38]. Briefly, semen samples were diluted (1:9) in an extender composed of 59 mM NaCl, 6.3 mM KCl, 0.68 mM CaCl_2_, 2.1 mM Mg_2_SO_4_, 27 mM NaHCO_3_, 3.4 mM sucrose, 69 mM D-mannitol, 118 mM Tris-HCl, pH 8.1 and 16% ethylene glycol. After dilution with the extender, the samples were loaded into 0.5-ml straws and kept in liquid nitrogen vapor, 3 cm above the surface of the liquid nitrogen, for 5 min. Then the straws were plunged into liquid nitrogen and storage for two weeks. After freezing straws were thawed by immersing them in a water bath (40°C) for 13 s.

The motility parameters of fresh and cryopreserved semen were determined by computer-assisted sperm analysis (CASA) using the two-step method for motility measurement, described by Rurangwa et al. [14]. A decrease in the percentage of sperm motility induced by cryopreservation was used as a marker for semen freezability. In a set of 20 male carp we arbitrary distinguished two groups with semen with high and low freezability. The six semen samples with the greatest decrease in sperm motility after freezing and thawing were classified as having PF (this yielded a threshold of ≥ 40% for the decrease in MOT sperm). The six semen samples with the smallest decrease were classified as having GF (threshold for the decrease in MOT sperm was ≤ 20%).

All sperm motion kinetic parameters measured before and after cryopreservation using the CASA system (VCL, VSL, VAP, PFT, MAD, BCF, ALH, DMN, LIN, STR, Mot) are presented in S1 Table. After cryopreservation the percentage of motile sperm was lower in PF semen than GF semen. The other sperm motion kinetics except STR did not differ between GF and PF. However in fresh sperm from both groups STR and other sperm motion parameters were similar. The results of four spermatic kinetics % MOT, VCL, VSL and ALH for both groups are presented in our previous study [37].

### Preparation of fresh spermatozoa and supernatant after cryopreservation from good and poor freezability carp semen

To obtain spermatozoa, good (n = 6) and poor (n = 6) freezability semen was centrifuged at 3,000× g for 30 min (4°C). After the removal of the supernatant, the pellets (containing the spermatozoa) were washed twice in a sperm immobilizing solution (20 mM Tris-HCl, 200 mM KCl, pH 8.0) by centrifugation at 3,000x g at 4°C for 30 min. Proteins were extracted from spermatozoa with lysis buffer (8 M urea, 2M thiourea, 4% [w/v] 3-[(3-cholamidopropyl)-dimethylammonio]-1-propanesulfonate [CHAPS]), 0.1% (w/v) Triton X-100, 100 mM dithiotreitol (DTT), 2% (v/v) immobilized pH gradient (IPG) buffer (3–10 NL), and 2.5% (v/v) protease inhibitor cocktail (Sigma-Aldrich, St. Louis, USA). The samples were sonicated (5 s three times), kept on ice for 1 h, and centrifuged for 10 min at 14,000× g at 4°C. The protein extract (spermatozoa) were stored at -80°C until analysis.

A supernatant after cryopreservation is defined as a fluid surrounding extended semen after cryopreservation. It is composed of seminal plasma, an extender and substances that leak from spermatozoa after freezing and thawing. To obtain supernatants after cryopreservation cryopreserved semen of good and poor freezability were centrifuged at 3,000× g for 30 min at 4°C, followed by centrifugation of the supernatant for 10 min at 12,000× g at 4°C. The supernatant of the cryopreserved semen was collected and stored at -80°C. Prior to proteomic analysis, the supernatants of GF and PF semen were concentrated using an Amicon ultracentrifuge (3 kDa molecular weight cut off [Millipore]).

Before proteomic analysis, aliquots containing approximately 800 μg of proteins (spermatozoa and supernatant after cryopreservation) were processed using a Clean-Up Kit (GE Healthcare, Uppsala, Sweden) according to the manufacturer’s protocol. Samples were resuspended in 80 μL of DIGE labeling buffer consisting of 30 mM Tris, 7 M urea, 2 M thiourea, and 4% CHAPS, to a protein concentration of 5–10 mg/mL. The protein concentration prior to and after the cleaning procedure was measured using a Coomassie Plus Kit (Thermo Scientific, Rockford, IL, USA) with bovine serum albumin as the standard.

### 2D-DIGE analysis of fresh spermatozoa and supernatant after cryopreservation of good and poor freezability semen

Two independent 2D-DIGE analysis were performed in order to compare i) the protein profiles of spermatozoa of GF and PF semen (n = 6 for each group), and ii) the protein profiles of supernatant after cryopreservation of GF and PF semen (n = 6 for each group). For each biological replicate, 50 μg of protein extract of each sample type (spermatozoa and supernatant after cryopreservation of GF and PF) was labelled with 400 pmol of Cy3 or Cy5 (GE Healthcare), respectively. A dye swap (Cy3/Cy5) was performed between good and poor freezability samples to exclude dye bias. An internal standard was created by mixing equal amounts of each sample within experiment and was labelled with Cy2. After incubation on ice and in the dark for 30 in, the reaction was terminated by adding 10 mM lysine. The three labeled samples were then combining within each experiment according to the scheme presented in S2 and S3 Tables and diluted with rehydration buffer (8 M urea, 2% CHAPS, 18 mM dithiothreitol (DTT), 0.5% carrier ampholyte, pH (3–10 NL) to 340 μl. The combined samples were loaded on a pH gradient strip (18 cm, pH 3–10 NL) for isoelectric focusing (IEF) on an Ettan IPGphor system (Amersham Bio-sciences, Uppsala, Sweden) as described by Dietrich et al. [39]. After IEF, the strips were first equilibrated in equilibration solution of 50 mM Tris–HCl (pH 8.8), 6 M urea, 30% (v/v) glycerol, 2% (w/v) SDS, traces of bromophenol blue, and 1% (w/v) DTT for 15 min, and later in the same solution except that DTT was replaced by 4% (w/v) iodoacetamide for a further 15 min. Equilibrated immobilized pH gradient (IPG) strips were transferred onto 12.5% vertical polyacrylamide gels (gel size 25.5 × 19.6 cm, 1-mm thickness, cast in low fluorescence glass plates using an Ettan Dalt six system [GE Healthcare, Uppsala, Sweden]). Electrophoresis was conducted overnight at 1.5 W per gel constant current. The Cy2-, Cy3-, and Cy5-labeled images were acquired on a Typhoon 9400 scanner (Amersham Biosciences) at excitation and emission values of 488/520, 532/580, and 633/670 nm, respectively.

Intragel spot detection and quantification and intergel matching and quantification were performed using the differential in-gel analysis (DIA) and biological variation analysis (BVA) modules of DeCyder software version 6.5 (Amersham Bio-sciences). During spot detection, the estimated number of spots was set at 10,000 and volume <30,000. The resulting spot maps were exported to BVA. Gel-to-gel matching of the standard spot maps from each gel, followed by statistical analysis of protein abundance change between samples, was performed in the BVA module. Selection criteria for the detection of significantly changed protein spots were that protein spots were detected in all of the analyzed gels with statistical significance (p ≤ 0.05).

### Protein digestion and mass spectrometry analysis

Differentially expressed protein spots of interest were excised from the DIGE gels and subjected to in-gel digestion with trypsin. Briefly, gel plugs were destained with 30% acetonitrile in 100 mM ammonium bicarbonate (NH_4_HCO_3_) for 30 min and vacuum dried. Then, digestion solution (20 ng/μl trypsin (Promega, Madison, WI, USA) in 20 mM NH_4_HCO_3_ was added, and the samples were digested at 37°C overnight. After digestion, peptides were extracted with 0.1% trifluoroacetic acid and then concentrated and desalted using Zip-Tip C18 pipette tips (Millipore), as described by Dietrich et al. [27]. Equal volumes of sample and α-HCCA matrix (5 mg/ml) were spotted and mixed on the MALDI-TOF target plate. Peptide mixtures were analyzed with a Bruker Daltonics AutoFlex TOF-TOF LIFT Mass Spectrometer (Bruker Daltonics, Bremen, Germany) in positive ion reflector mode. The accelerating potential was 20 kV with eight shots per second. Each spectrum was internally calibrated using monoisotpoic [M^+^H]^+^ ion peptide calibration standards (Bruker Daltonics) consisting of Angiotensin II (1046.54), Angiotensin I (1296.68), Substance P (1347.73), Bombesin (1619.82), ACTH clip 1 (2093.086), ACTH clip 18 (2465.19), and Somatostatin 28 (3147.471). The MS peptide mass fingerprint (PMF) and fragment mass spectra (MS/MS) from each individual spot were combined and used to search against a NCBInr Bony Fishes database (a database containing 3,005,095 proteins was generated on the 30^th^ January 2017 using MascotServer [Matrix Sciences]) with the following settings: cleavage enzyme, trypsin; max missed cleavages 2; mass tolerance mono, 50 ppm; fragment ion mass tolerance, 0.5 Da; parent ion mass tolerance, 200 ppm; alkylation of cysteine by carbamidomethylation as a fixed modification; and oxidation of methionine as a variable modification. For the PMF and MS/MS ion search, statistically significant (*p* ≤ 0.05) matches by MASCOT were regarded as correct hits. Proteins identified as hypothetical were searched in terms of protein sequence similarity using the Basic Local Alignment Search Tool (BLAST).

### Ingenuity pathway and STRING analyses

To understand the biological context of the identified proteins and their involvement in biological pathways, the differentially abundant proteins were subjected to ingenuity pathway analysis (IPA) classification (version 9.0, http://www.pantherdb.org/). Because IPA only accepts gene or protein accession numbers representing human, mouse and rat genes or proteins, orthologs of the identified carp proteins belonging to those three species were first identified, and the accession numbers of the top blast hits were uploaded to IPA. Each identifier was associated with the IPA knowledge base and used to generate networks and to identify the proteins of the top five categories for each of the functional domains and canonical pathways. Fisher’s exact test and Benjamini-Hochberg multiple testing corrections were used to calculate the significance (*p* ≤ 0.05) of functional and canonical pathways of proteins involved in sperm motility.

For protein–protein interaction network analysis, the differentially expressed proteins (as determined by BVA) were analyzed using the STRING database version 10.5 (Search Tool for the Retrieval of Interacting Genes, http://string-db.org/) with a high confidence score (score > 0.7). The UniProt accession number for all of the identified proteins was submitted and mapped against a reference *Homo sapiens* dataset. The interaction networks were obtained based on confidence and evidence.

### Validation of 2D-DIGE results by Western blotting

We used antibodies against carp Pv produced in our laboratory by immunization of rabbit with Pv isolated from carp spermatozoa [40] to confirm the identification of Pv as a protein whose abundance varies with semen freezability. Aliquots (100 μg) of spermatozoa proteins from GF and PF semen (both n = 6) G (n = 6) made up to 125 μl with rehydration buffer were applied to 7-cm IPG strips (pH range 4–7; GE Healthcare). Isoelectric focusing was performed according to the manufacturer’s protocol (GE Healthcare), and then the IPG strips were equilibrated with 2% dithiothreitol and 2.5% iodoacetamide. Each strip was laid onto a 15% SDS-PAGE gel (10 x 8 x 0.1 cm) for second dimension electrophoresis. Western blots were performed as described by Dietrich et al. [39]. Briefly, six samples of spermatozoa from GF and PF semen were transferred to nitrocellulose membranes after 2DE. The membranes were incubated overnight at 4°C with anti-Pv antibodies diluted 1:20,000 in TBS-T (0.05 M Tris-HCl, 0.15 M NaCl, and 0.1% Tween 20, pH 7.6). After rinsing to remove unbound primary antibodies, the membranes were incubated with alkaline-phosphatase -(AP)-conjugated horse anti-rabbit antibodies (Sigma) diluted 1:20,000 in TBS-T for two hours at room temperature. Products were visualized by incubation with 5-bromo4-chloro-30-indolyphosphate and nitro-blue tetrazolium (BCIP/ NBT) reagent solvent in the dark for 10 min, after which the membranes were washed with 2 mM EDTA to stop the color reaction. Blots were then scanned with the VersaDoc MP 4000 system (Bio-Rad). Using Quantity One Analysis Software, version 4.6.9 (Bio-Rad), Pv spots were quantified by Density/Area (INT/mm2). The relative abundance of Pv was calculated as the ratio of single spot abundance to the total abundance of Pv.

## Results

### Comparison of fresh spermatozoa proteome of good and poor freezability semen

**Fig 1:**
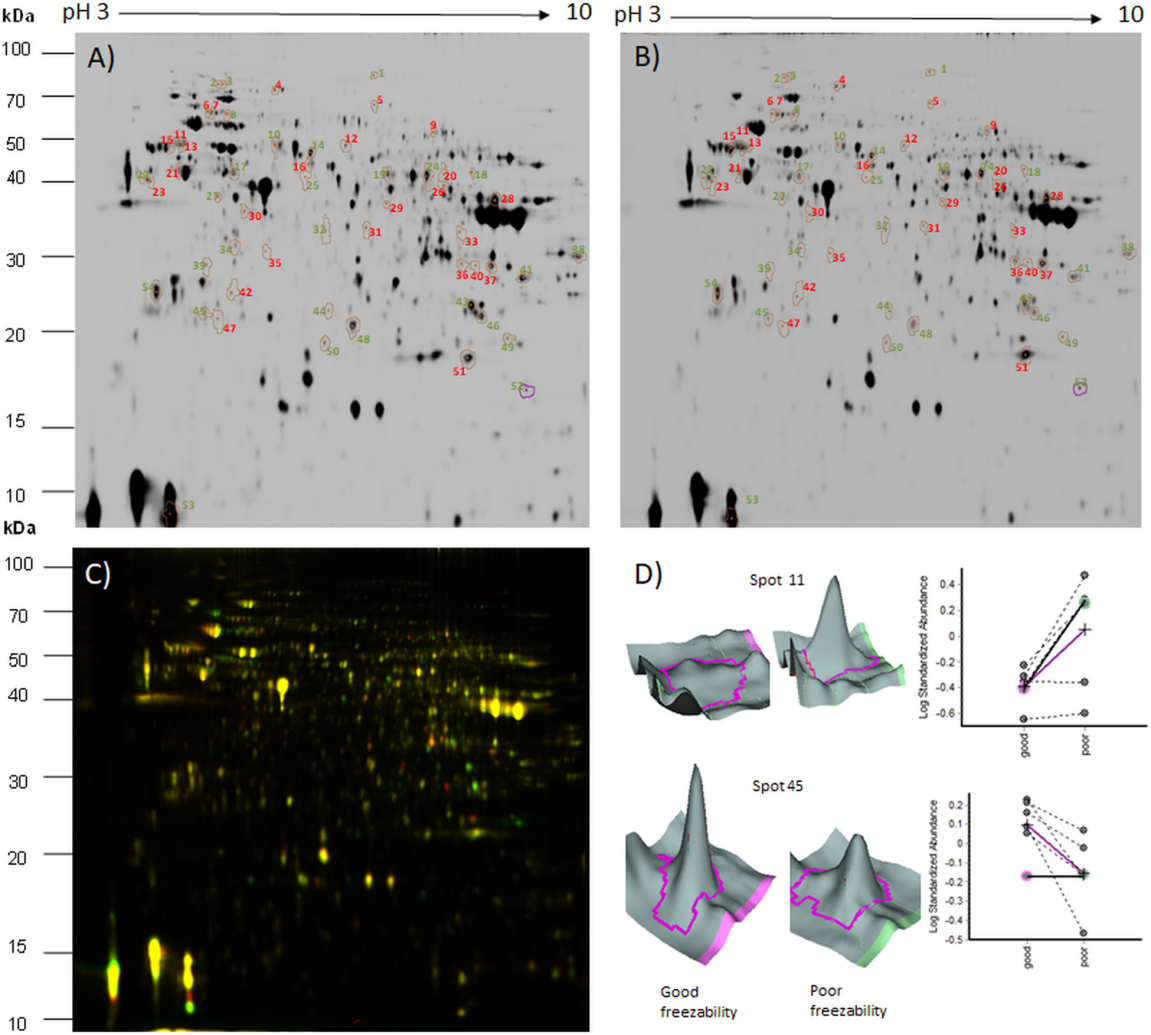
The representative pattern of spermatozoa proteomes of good (A) and poor (B) freezability semen after 2D-DIGE analysis. C-overlay of spermatozoa of good (GF) and poor (PF) semen (Cy5 [red]—spermatozoa of PF; Cy3 [green]—spermatozoa of GF). Numbers indicate spots with significantly different abundances between the spermatozoa of the GF and PF groups (red numbers indicate proteins more abundant in spermatozoa of PF, green numbers indicate proteins more abundance in spermatozoa of GF semen). (C)- the 3-D (representative images) and line charts (data for six males) of the chosen spots (spot 11- protein kinase C and casein kinase substrate in neurons protein 2 and spot 45—meichroacidin-like sperm-specific axonemal protein).

The analysis of spermatozoa proteins from good and poor freezability semen using quantitative 2D-DIGE technology led to the detection of 1,555 matched spots, of which 54 spots had significantly difference abundances (>1.1-fold change, p<0.05) between GF and PF (Fig 1A and 1B). Of the differentially expressed spots, 28 spots were upregulated in spermatozoa of GF semen of which 22 were successfully identified by MALDI-TOF/TOF (Table 1). Twenty-six spots were found to be upregulated in spermatozoa of PF semen and 22 were successfully identified as 18 unique proteins (Table 1). The overlay of spermatozoa proteins of GF and PF males is shown in Fig 1C. The Fig 1D shows 3-dimensional images and line charts representing the relative quantification of differentially abundant protein spots between spermatozoa of GF and PF males.

**Table 1:** Proteins of carp spermatozoa differentially expressed between good and poor freezability semen.

Spot no in Fig 1.	Protein name/organism	Gene name	Gi number	Protein score	% sequence coverage	# of peptides (ion score >30)	MW/pI	Good/Poor ratio	Poor/Good ratio	P value
***Proteins more abundant in spermatozoa of good freezability semen***										
8	dipeptidyl peptidase 3-like [*Cyprinus carpio*]	Dpp3	XP_018929389.1	123	16	2	64.3/5.3	1.2		0.047
10	PREDICTED: aldehyde dehydrogenase family 9 member A1-A-like [*Sinocyclocheilus anshuiensis*]	ALDH9A1	XP_016350584.1	293	16	3	56.1/5.7	1.3		0.008
14	PREDICTED: protein disulfide-isomerase A3-like [*Sinocyclocheilus anshuiensis*]	PDIA3	XP_016352018.1	423	31	6	55.1/5.1	1.2		0.023
17	PREDICTED: protein disulfide-isomerase A6-like [*Cyprinus carpio*]	Pdia6	XP_018981098.1	731	31	7	48.3/5.3	1.5		0.034
19	PREDICTED: bleomycin hydrolase-like [*Cyprinus carpio*]	Blmh	XP_018958153.1	247	41	3	40.2/9.1	1.5		0.048
24	PREDICTED: adenosylhomocysteinase B [*Sinocyclocheilus grahami*]	AHCY	XP_016092109.1	168	44	3	48.5/6.3	1.4		0.044
25	PREDICTED: dihydrolipoyllysine-residue succinyltransferase component of 2-oxoglutarate dehydrogenase complex, mitochondrial-like [*Fundulus heteroclitus*]	Dlst	gi|831475342	117	12	2	49.6/8.5	1.4		0.021
27	PREDICTED: mannose-6-phosphate isomerase isoform X1 [*Sinocyclocheilus rhinocerous*]	Mpi	XP_016389137.1	172	23	3	51.6/6.0	1.3		0.018
32	PREDICTED: prostaglandin reductase 2 [*Cyprinus carpio*]	PTGR2	XP_018951150.1	189	26	3	27.2/5.4	1.2		0.0088
34	PREDICTED: glutaredoxin-3 [*Sinocyclocheilus grahami*]	Glrx3	XP_016123412.1	163	25	3	18.9/5.6	1.3		0.047
38	voltage-dependent anion-selective channel protein 3-like [*Scleropages formosus*]	VDAC3	KPP76017.1	263	26	3	30.4/8.3	1.5		0.032
39	PREDICTED: glycerol-3-phosphate phosphatase-like [Sinocyclocheilus anshuiensis]	PGP	XP_016316835.1	199	24	2	33.9/5.6	1.2		0.0045
41	PREDICTED: proteasome subunit alpha type-4 isoform X1 [Cyprinus carpio]	Psma4	XP_018930832.1	793	75	8	29.6/8.1	1.2		0.0096
43	glutathione S-transferase rho [*Cyprinus carpio*]	GSTZ1	BAS29983.1	576	55	7	26.6/7.0	1.4		0.02
45	Meichroacidin-like sperm-specific axonemal protein [*Cyprinus carpio*]	Rsph1	Q6VTH5.1	275	23	3	24.7/5.1	1.6		0.03
46	PREDICTED: proteasome subunit beta type-7-like [*Sinocyclocheilus anshuiensis*]	Psmb7	XP_016359595.1	348	23	5	30.2/8.4	1.2		0.034
48	proteasome subunit alpha type-2 [*Astyanax mexicanus*]	PSMA2	XP_007258904.1	552	59	4	25.8/6.0	1.2		0.032
49	Pi-class glutathione S-transferase [*Cyprinus carpio*]	GSTP1	gi|112901122	234	34	2	23.9/7.6	1.3		0.019
50	PREDICTED: proteasome subunit beta type-4-like [*Cyprinus carpio*]	Psmb4	XP_018975946.1	264	20	3	28.6/6.0	1.2		0.0046
52	PREDICTED: proteasome subunit beta type-5-like [*Cyprinus carpio*]	Psmb5	XP_018940253.1	514	39	6	29.9/5.6	1.4		0.02
53	pvalb6 protein [*Cyprinus carpio*]	PVALB	gi|527816862	320	62	4	12.2/4.9	1.3		0.017
54	PREDICTED: proteasome subunit alpha type-5 [*Cyprinus carpio*]	Psma5	XP_018966460.1	590	68	5	26.6/4.7	1.2		0.0024
***Proteins more abundant in spermatozoa of poor freezability semen***										
4	PREDICTED: neutral alpha-glucosidase AB-like [*Sinocyclocheilus rhinocerous*]	GANAB	XP_016429124.1	704	24	9	106.7/5.4		1.2	0.031
5	PREDICTED: methylmalonyl-CoA mutase, mitochondrial-like isoform X1 [*Sinocyclocheilus rhinocerous*]	MUT	XP_016391928.1	840	37	9	84.3/6.8		2.8	0.048
6	PREDICTED: dipeptidyl peptidase 3-like [*Cyprinus carpio*]	Dpp3	XP_018929391.1	309	36	3	67.1/5.2		2.0	0.03
7	PREDICTED: dipeptidyl peptidase 3-like isoform X3 [*Sinocyclocheilus anshuiensis*]	Dpp3	XP_016317008.1	384	21	3	82.5/5.1		1.3	0.011
9	PREDICTED: transketolase-like [S*inocyclocheilus anshuiensis*]	TKT	XP_016320493.1	115	12	2	68.6/6.8		1.3	0.017
11	PREDICTED: protein kinase C and casein kinase substrate in neurons protein 2 [*Cyprinus carpio]*	Pacsin2	XP_018935227.1	368	27	4	50.9/5.4		1.9	0.0002
12	PREDICTED: phosphoglucomutase-1 [*Cyprinus carpio*]	Pgm1	XP_018950613.1	824	48	8	61.4/5.8		1.7	0.026
13	PREDICTED: protein kinase C and casein kinase substrate in neurons protein 2 [*Cyprinus carpio*]	PACSIN2	XP_018935227.1	379	25	4	50.9/5.4		1.6	0.0001
15	PREDICTED: protein disulfide-isomerase A3-like [*Cyprinus carpio*]	PDIA3	XP_018960961.1	828	46	10	55.4/4.8		1.7	0.0062
16	PREDICTED: aldehyde dehydrogenase, mitochondrial [*Sinocyclocheilus grahami*]	Aldh2	XP_016084728.1	92	13	2	56.7/6.9		1.1	0.042
20	PREDICTED: adenosylhomocysteinase B [*Sinocyclocheilus grahami*]	AHCY	XP_016092109.1	170	20	2	48.5/6.3		3.6	0.037
26	phosphogluconate dehydrogenase [*Danio rerio*]	Pgd	gi|37362266	205	22	2	53.7/6.3		1.2	0.012
28	PREDICTED: isocitrate dehydrogenase [NADP], mitochondrial [*Stegastes partitus*]	Idh2	gi|657582263	496	43	5	51.1/7.9		1.1	0.031
30	PREDICTED: transaldolase-like [*Sinocyclocheilus grahami*]	TALDO1	XP_016146919.1	168	23	3	37.9/5.6		1.3	0.0006
31	PREDICTED: S-formylglutathione hydrolase isoform X1 [*Astyanax mexicanus*]	Esd	gi|597747154	167	21	2	31.6/5.7		1.2	0.037
33	PREDICTED: glyoxylate reductase/hydroxypyruvate reductase [*Cyprinus carpio*]	GRHPR	XP_018956524.1	129	26	2	29.4/9.2		1.6	0.015
36	PREDICTED: malate dehydrogenase, mitochondrial isoform X2 [*Cyprinus carpio*]	Mdh2	XP_018957873.1	895	50	8	35.8/8.6		1.5	0.009
37	PREDICTED: malate dehydrogenase, mitochondrial isoform X2 [*Cyprinus carpio*]	MDH2	XP_018957873.1	1230	66	10	35.8/8.6		1.7	0.018
42	PREDICTED: 6-phosphogluconolactonase isoform X1 [*Danio rerio*]	PGLS	gi|528475142	119	22	2	27.2/5.5		1.2	0.019
47	Meichroacidin-like sperm-specific axonemal protein [*Cyprinus carpio*]	Rsph1	gi|60415990	302	32	3	24.7/5.1		1.5	0.007
51	PREDICTED: high mobility group protein B3-like [*Sinocyclocheilus grahami*]	Hmgb3	XP_016123094.1	400	33	4	23.2/5.9		1.5	0.01

### Comparison of supernatant after cryopreservation of good and poor freezability semen

**Fig 2:**
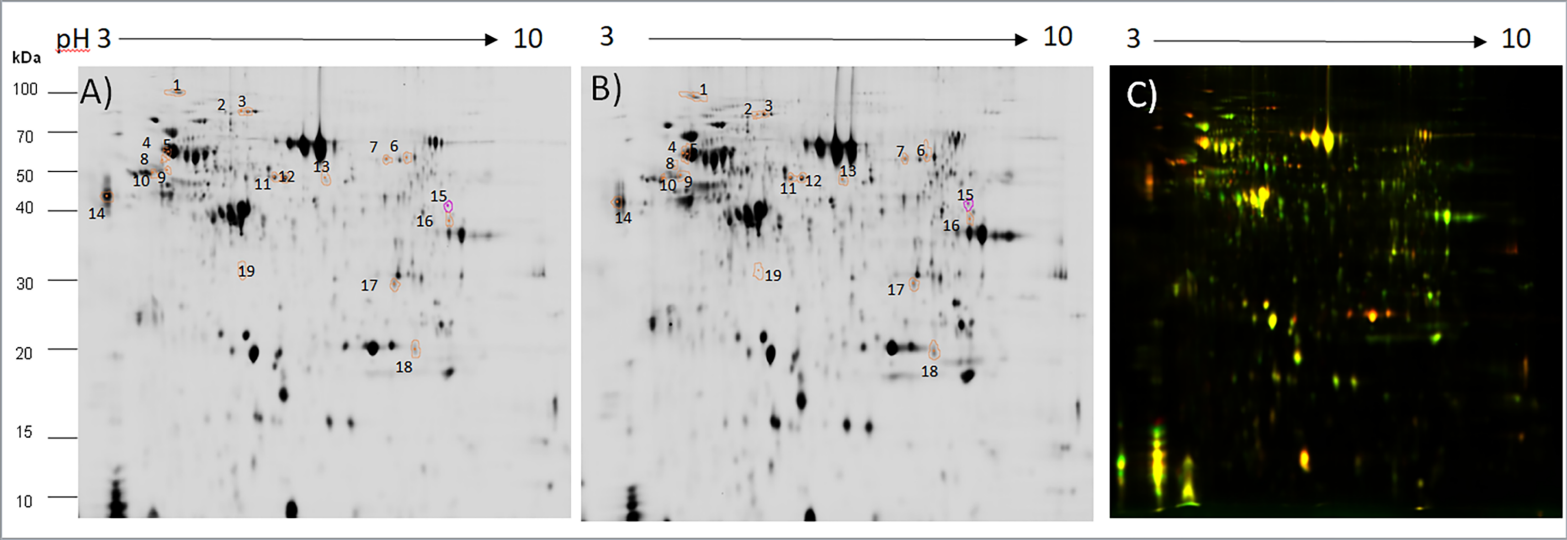
The representative pattern of the supernatant after cryopreservation of good (Cy3, green spots) and poor freezability (Cy5, red spots) semen after 2D-DIGE analysis. Numbers indicate proteins more abundance in the supernatant after cryopreservation of poor freezability semen.

The analysis of supernatant after cryopreservation of GF and PF semen using 2D-DIGE led to detection of 1,616 matched spots. Of these, 19 were more abundant (p<0.05) in supernatant of PF semen than GF semen (Fig 2A and 2B). Eighteen of these spots were successfully identified as 12 unique proteins (Table 2). A representative 2D-DIGE image of an overlay of supernatant after cryopreservation of GF and PF semen is shown in Fig 2.

**Table 2:** Proteins more abundant in the supernatant after cryopreservation of poor freezability semen.

Spot no in Fig 2.	Protein name / organism	Gene name	Gi number	Protein score	% sequence coverage	# of peptides (ion score >30)	Poor/Good ratio	P value
1	PREDICTED: hypoxia up-regulated protein 1-like [*Sinocyclocheilus rhinocerous*]	Hyou1	XP_016416870.1	529	27	5	1.4	0.036
2	PREDICTED: neutral alpha-glucosidase AB-like [*Sinocyclocheilus rhinocerous*]	GANAB	XP_016429124.1	324	22	5	1.4	0.029
3	PREDICTED: neutral alpha-glucosidase AB-like [*Sinocyclocheilus rhinocerous*]	GANAB	gi|1025212462	400	30	4	1.5	0.015
4	PREDICTED: 78 kDa glucose-regulated protein [*Sinocyclocheilus graha*mi]	GRP78	gi|1020548928	760	31	5	1.5	0.015
5	PREDICTED: 78 kDa glucose-regulated protein [*Sinocyclocheilus grahami*]	GRP78	gi|1020548928	680	31	5	1.4	0.03
6	PREDICTED: transketolase-like [*Sinocyclocheilus rhinocerous*]	TKT	XP_016373536.1	507	24	4	1.3	0.047
7	PREDICTED: EH domain-containing protein 1-like [*Sinocyclocheilus anshuiensis*]	EHD1	gi|1025175231	432	47	5	1.2	0.0082
8	PREDICTED: protein kinase C and casein kinase substrate in neurons protein 2 [*Cyprinus carpio*]	PACSIN2	XP_018935227.1	164	15	2	1.9	0.0063
9	protein kinase C and casein kinase substrate in neurons protein 1 [*Mesocricetus auratus*]	PACSIN2	XP_005084936.1	149	18	2	1.5	0.0075
10	PREDICTED: protein disulfide-isomerase A3-like [*Cyprinus carpio*]	PDIA3	XP_018960961.1	393	32	5	2.0	0.0085
11	PREDICTED: protein disulfide-isomerase A3-like [*Sinocyclocheilus anshuiensis*]	PDIA3	XP_016352018.1	377	27	4	1.4	0.022
12	PREDICTED: protein disulfide-isomerase A3 [*Cyprinus carpio*]	PDIA3	XP_018939030.1	534	46	7	1.5	0.019
13	PREDICTED: protein disulfide-isomerase A3-like [*Sinocyclocheilus rhinocerous*]	PDIA3	XP_016426895.1	166	20	2	1.3	0.01
14	calreticulin [*Lates calcarifer*]	CALR	ADQ92841.1	355	15	4	1.3	0.05
15	PREDICTED: elongation factor 1-gamma-like [*Cyprinus carpio*]	EEF1G	XP_018943137.1	530	32	6	1.2	0.022
16	PREDICTED: isocitrate dehydrogenase [NADP], mitochondrial [*Pygocentrus nattereri*]	IDH2	XP_017566604.1	220	37	2	1.2	0.038
17	PREDICTED: L-lactate dehydrogenase B-A chain-like [*Sinocyclocheilus grahami*]	Ldhb	XP_016134056.1	639	36	7	1.7	0.026
18	GTP-binding nuclear protein Ran [*Osmerus mordax*]	RAN	gi|225708424	348	47	4	1.2	0.028

### Ingenuity pathway analysis of the differentially abundant proteins

A total of 22 and 18 proteins overexpressed in spermatozoa of GF and PF semen, respectively and 12 proteins present in higher abundance in supernatant after cryopreservation of PF semen were analyzed by IPA, in terms of their canonical signal pathway, molecular and cellular function, and networks function. A summary of all associated pathways and functions for the differentially abundant proteins is presented in Table 3. The top canonical pathways associated with proteins of higher abundance in sperm of GF were protein ubiquitination pathway, antigen presentation pathway, cell redox homeostasis, D-mannose degradation and L-carnitine biosynthesis while the molecular and cellular functions including amino acid metabolism, small molecule biochemistry, cell death and survival, cell morphology, and cellular assembly and organization.

**Table 3:** IPA analysis overview of differentially abundant sperm proteins of good and poor.

**Top canonical pathways**	**No of molecules**	**Proteins**
***Proteins more abundant in spermatozoa of good freezability semen***		
Protein ubiquitination pathway	6	PSMA1, PSMA4, PSMA5, PSMB4, PSMB5, PSMB7
Antigen presentation pathway	8	PSMA1, PSMA4, PSMA5, PSMB4, PSMB5, PSMB7, BLMH, PDIA3
Cell redox homeostasis	2	GLRX3, PDIA6
D-mannose degradation	1	MPI
L-carnitine biosynthesis	1	ALDH9A1
***Proteins more abundant in spermatozoa of poor freezability semen***		
Pentose phosphate pathway	5	PGD, PGLS, TALDO1, TKT, PGM2
Superpathway of methionine degradation	2	AHCY, MUT
***Proteins more abundant in supernatant of poor freezability semen***		
Endoplasmic reticulum stress pathway	4	CALR, HSPA5, PDIA3, HYOU1
Antigen processing and presentation	3	CALR, PDIA3, HSPA5
Protein processing in endoplasmic reticulum	5	CALR, PDIA3, GANAB, HSPA5, HYOU1
Glutathione redox Reactions	1	PDIA3
**Top molecular and cellular functions**	**No of molecules**	**Proteins**
***Proteins more abundant in spermatozoa of good freezability semen***		
Amino acid metabolism	4	AHCY, BLMH, GSTZ1, PVALB
Small molecule biochemistry	10	DLST, VDAC3, PGP, PDIA3, ALDH9A1, AHCY, BLMH, GSTZ1, PVALB, MPI
Cell death and survival	6	PSMA4, PSMA5, PSMB5, PSMB4, DP3, PDIA3
Cell morphology	4	PVALB, VDAC3, PDIA3, RSPH1
Cellular assembly and organization	3	PVALB, VDAC3, RSPH1
***Proteins more abundant in spermatozoa of poor freezability semen***		
Carbohydrate metabolism	9	PGD, PGLS, TALDO1, TKT, ALDH2, PGM1, IDH2, PACSIN2, GANAB
Oxidative reduction process	9	PGM1, GRHPR, MUT, ALDH2, IDH2, TKT, TALDO1, PGD, PDIA3
Cellular aldehyde metabolism	5	GRHPR, ESD, IDH2, PGD, TKT
Cellular movement	5	ALDH2, PDIA3, TKT, AHCY, HMGB3
***Proteins more abundant in supernatant of poor freezability semen***		
Cellular movement	7	HSPA5, PDIA3, TKT, HYOU1, CALR, PACSIN2, EHD1
Response to endoplasmic reticulum stress	4	CALR, HSPA5, RAN, HYOU1
Cellular function and maintenance	7	CALR, HSPA5, EDH1, HYOU1, PACSIN2, PDIA3, RAN
Molecular transport	7	CALR, EDH1, PDIA3, RAN, HSPA5, HYOU1, GANAB
**Functions associated with network**		**Score**
***Proteins more abundant in spermatozoa of good freezability semen***		
Cell morphology, Energy production, molecular transport		24
***Proteins more abundant in spermatozoa of poor freezability semen***		
Carbohydrate metabolism, nucleic acid metabolism, small molecule metabolism		50
***Proteins more abundant in supernatant of poor freezability semen***		
Carbohydrate metabolism, molecular transport, small molecule metabolism		32

IPA demonstrated pentose phosphate pathway and superpathway of methionine degradation as the canonical pathways most affected by proteins of higher abundance in PF semen. These proteins were associated with carbohydrate metabolism, an oxidative reduction process, cellular aldehyde metabolism, and cellular movement.

The proteins that were enriched in the supernatant after cryopreservation of PF semen were involved in the endoplasmic reticulum stress pathway, antigen processing and presentation, and protein folding. The molecular and cellular functions of these proteins were associated with cellular movement, response to endoplasmic reticulum stress, cellular function and maintenance, transport and protein trafficking.

### Protein-protein interaction network analysis of differentially abundant proteins from good and poor freezability semen

**Fig 3:**
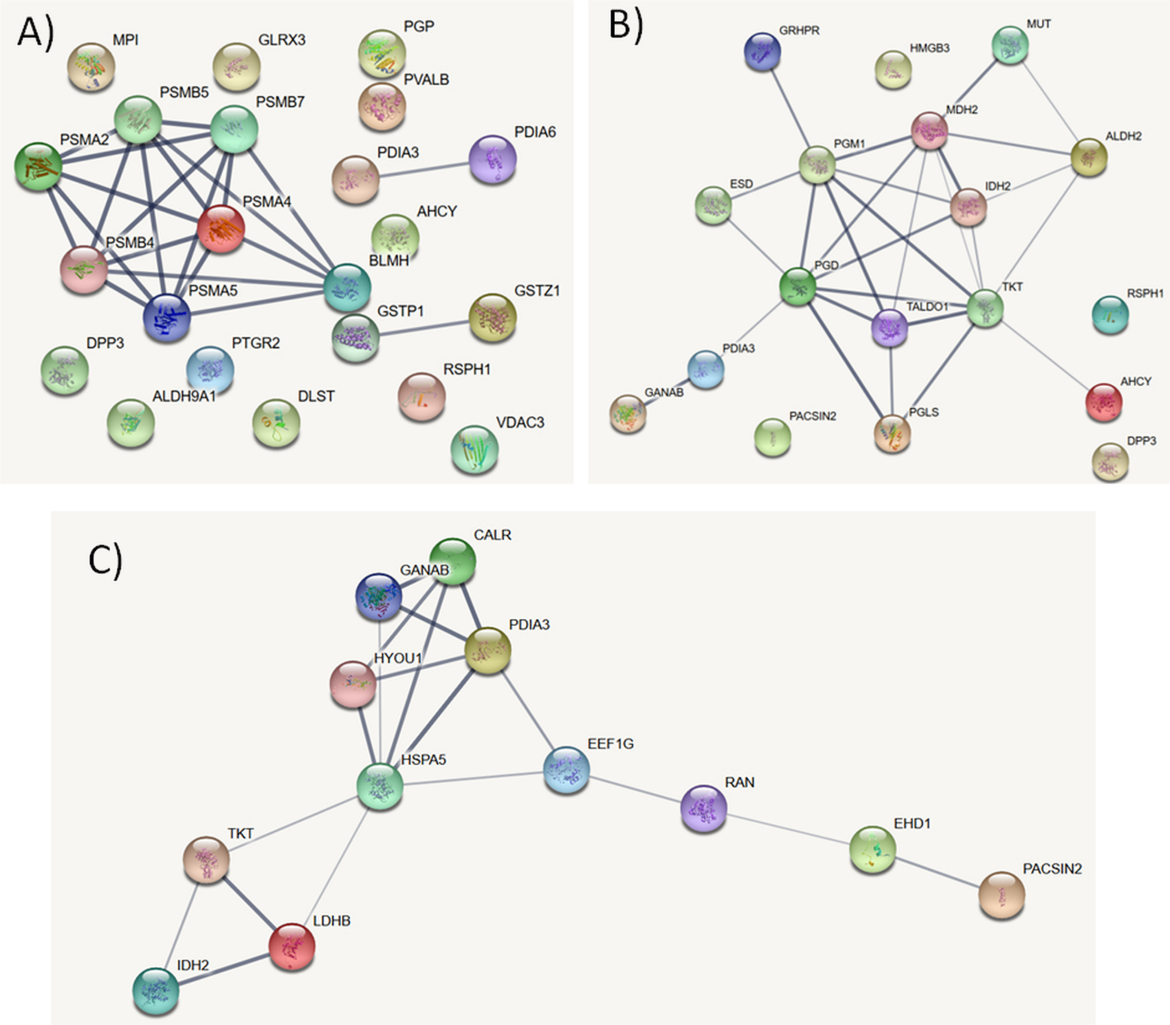
Protein-protein interaction networks of spermatozoa proteins differentially abundant between good (GF) and poor freezability (PF) semen. Different clusters of interacting proteins were identified using the STRING software with a high confidence score. (A) Sperm proteins with higher abundance in GF. (B) Sperm proteins with higher abundance in PF. (C) Proteins enriched in the supernatant after cryopreservation of PF. The line size indicates a high interaction score (tight lines indicate high score > 0.9; thin lines indicate a medium score > 0.7). The proteins identified in carp spermatozoa of GF and PF males and supernatant after cryopreservation are shown in Table 1 and Table 2, respectively.

We performed an interaction network analysis of those proteins enriched in spermatozoa of GF semen using STRING. We found that, of the 22 proteins, 11 proteins interact with each other (22 edges). The most connected proteins (clustering coefficient, 0.983; PPI enrichment p-value, 4.33e-15) are involved in proteasomal ubiquitin, protein catabolic process, and antigen processing and presentation (Fig 3A). For the 18 proteins enriched in spermatozoa of PF we detected 28 edges (14 proteins interacted with each other), with a clustering coefficient of 0.789 (enrichment p-value: 4.33e-16). These proteins were involved in oxidative reduction process and cellular aldehyde metabolic process (Fig 3B).

Furthermore, STRING analysis of the proteins enriched in supernatant after cryopreservation of PF semen, demonstrated that all 12 proteins were connected (19 edges, clustering coefficient of 0.619, PPI enrichment p-value of 1.61e-13). The most connected proteins were involved in protein folding in the endoplasmic reticulum, carbohydrate metabolic processes, and response to endoplasmic reticulum stress.

### Validation of 2D-DIGE by Western blot analysis of parvalbumin

**Fig 4:**
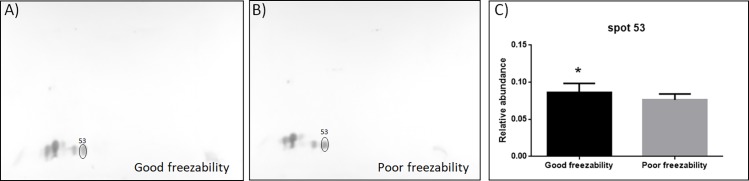
Validation of 2D-DIGE results by Western blotting using anti-parvalbumin (Pv) antibodies. Representative Western blot of spermatozoa of good freezability (GF) semen (A) and poor freezability (PF) semen (B). Proteins were separated by 2DE, transferred to nitrocellulose membranes and incubated with anti-Pv antibodies. Blots were scanned with the VersaDoc MP 4000 system (Bio-Rad, Hercules, CA). Spots indicate proteoforms of Pv detected using anti-Pv antibodies in spermatozoa of GF and PF. Number 53 indicate Pv isoform differing in abundance between GF and PF semen and correspond to spot 53 in Fig 1. Intensity/area (INT/mm2) of Pv proteoforms in the spermatzoa of GF semen (n = 6) and PF semen (n = 6) was quantified using Quantity One Analysis Software (Bio-Rad, Hercules, CA). (C) Relative abundance of Pv was calculated as a ratio of spot abundance in relation to the total abundance of Pv. The data shown are means ± SEM. Asterisks indicate significant differences between spermatozoa of GF and PF carp males (*p ≤ 0.05).

Polyclonal antibodies against carp parvalbumin detected four spots (proteoforms) of Pv (Fig 4A and 4B). As with the proteomic data, an increase (p ≤ 0.05) in the relative abundance of spot 53 (defined as ratio of spot abundance to total Pv abundance) was observed in the spermatozoa from GF semen (relative abundance 0.104) relative to spermatozoa from PF semen (relative abundance 0.084; Fig 4C). The Pv proteoform (spot 53) correspond to spot 53 identified as enriched in GF semen through 2D-DIGE (Fig 1). The abundance of the rest of the Pv isoforms and total Pv abundance was similar in GF and PF semen. Fig 4 shows a representative Pv protein spot pattern of from the Western blot analysis.

## Discussion

To our knowledge, this study is the first to present differentially expressed proteins between spermatozoa of good and poor freezability fish semen. Using a 2D-DIGE approach coupled with MALDI-TOF/TOF, comparing spermatozoa of GF and PF semen, we identified differentially expressed proteins. IPA analysis of these sperm proteins revealed distinct canonical pathways for GF and PF semen. We also identified 12 proteins enriched in the supernatant after cryopreservation of poor freezability semen. The obtained results should be valuable for better understanding of the mechanism of sperm freezability in fish especially for carp.

Among proteins enriched in spermatozoa of good freezability String analysis revealed multiple significant interaction between three α- and three β-type subunits of the 20S proteasome and bleomycin. These proteins creates one major cluster functionally involved in the ubiquitin proteasome pathway. The 26S proteasome plays a crucial role in the control of mitosis and meiosis and is responsible for protein degradation and cellular remodeling during spermatogenesis [41]. In mammals, the 26S proteasome complex has been shown to be more abundant in mature sperm than immature sperm [42], indicating its important roles in sperm motility and fertilization related events [43]. Recently, the 26S proteasome was identified as a positive marker of sperm freezing resilience in ram seminal plasma [44]. Blmh was identified for the first time in fish spermatozoa. Its function in mammals is related to antigen presentation, protein polyubiquitination, antioxidant defenses, response to toxic substances and sperm maturation [45]. Taken together, these findings suggest that the greater abundance of the proteasome and Blmh in the spermatozoa of GF might reflect the full maturation status of these spermatozoa, resulting in a greater resistance to cryoinjuries. The lower abundance of these proteins in PF spermatozoa could indicate incomplete maturation or aging phenomenon. Further studies should focus on analysis of the relationship between proteome changes and sperm maturation and aging.

Voltage-dependent anion selective channel protein (VDAC) is enriched in the spermatozoa of GF. VDAC is a pore forming protein, which, in mammalian spermatozoa, forms a channel structure in the lipid bilayer that mediates transport of ions and small molecules (Ca^2+^, Cl^-^, HCO_3_^-^, ATP, glutamate) through the outer mitochondrial membrane and plasma membrane [46, 47] an is also involved in the maintenance of flagellar structures of mature spermatozoa [48]. Moreover VDAC have been reported to be involved in spermatogenesis, sperm maturation, capacitation, acrosome reaction, the modulation of sperm motility [49, 50]. Vilagran et al. [24] identified VDAC2 as a potential positive marker of semen freezability in boars, which is in line with our findings. However, the role of VDAC in fish spermatozoa remains unknown. The presence of VDAC3 in higher amounts in spermatozoa of GF suggest its involvement in the protection of sperm from changes in membrane fluidity, through a better regulation of ion transmembrane flux during cold shock events, such that takes place during cryopreservation.

Among the proteins enriched in GF semen we identified enzymes important for protection against oxidative stress, including two forms (Rho and Pi) of glutathione S-transferase (GST), 4-trimethylaminobutyraldehyde dehydrogenase (ALDH9A1), and glutaredoxin. Li et al. [51] suggest that the cryopreservation of carp sperm induces oxidative stress in spermatozoa, demonstrated by higher levels of lipid peroxidation, protein oxidation, and glutathione peroxidase activity. Therefore, an enrichment of defensive intracellular and plasma membrane enzymes in spermatozoa of GF would be a great asset, as these cells are exposed to ROS generated during cryo-stress and their function could be associated with better protection of sperm membrane and proteins against oxidative damage occurring during cryopreservation.

Spermatozoa with good freezability had higher amount of proteins involved in cellular redox and energy metabolism, including dihydrolipoyllysine-residue succinyltransferase (DLST), glycerol-3-phosphate phosphatase, and mannose-6-phosphate isomerase. These two latter enzymes were not previously identified in carp spermatozoa and their function in energy production of spermatozoa. DLST is a component of the mitochondrial 2-oxoglutarate dehydrogenase complex. Recently, another component of this complex (dihydrolipoyl dehydrogenase) was found to be highly expressed in highly freezable bull sperm [28]. Taken together, our results suggest that the spermatozoa of GF are metabolically superior and have an advantage in energy storage (required for sperm motility) compared to spermatozoa of PF.

Parvalbumin, a Ca^2+^- binding protein, is present in multiple proteoforms in carp semen. Parvalbumin is a major protein of carp spermatozoa, which appears during the final step of spermatogenesis [40]. One proteoform of parvalbumin was found to be positively associated with carp semen cryopreservation. The crucial role of specific proteoforms of parvalbumin has been shown in mechanisms controlling carp sperm movement [39]. Since Ca^2+^ plays a pivotal role in carp sperm motility [52], the parvalbumin enriched in GF semen might be involved in the control of the Ca^2+^ gradient during sperm motility after cryopreservation. This is prerequisite both for control premature activation and activation of sperm motility. Taken together, these findings suggest that efficient control of Ca^2+^ gradient and sperm motility are beneficial for cryoresistance of carp spermatozoa.

Beside detection of proteins positively associated with freezability we also detected four proteins which proteoforms were differentially (positively or negatively) related to carp sperm freezability. Meichroacidin-like sperm-specific axonemal protein (MSAP) is a structural component of flagellar axoneme in carp and is expressed during late spermiogenesis and accumulated in mature spermatozoa, localized to basal body and flagellum of sperm [53]. MSAP function is associated with flagellar assembly and differentiation during spermiogenesis. Particular proteoforms of MSAP might produce slight differences in organization and stabilization of flagella structure, which can influence the resistance of sperm flagellum to cryopreservation stress.

Beside MSAP the other three proteins differentially related to carp sperm freezability were enzymes such as: dipeptidyl peptidase 3 (DPP3), protein disulfide isomerase A3-like (PDIA3), and adenosylhomocysteinase (AHCY). DPP3 and PDI are a part of a quality-control system for the correct folding of the proteins. PDI also functions as a molecular chaperone that prevents the formation of protein aggregates. AHCY is involved in the energetic metabolic pathway, leading to the production of phosphocreatine, which is essential for carp sperm motility. Thus, we propose that the freezability of carp sperm is related to proper protein folding and energy supply. The mechanism by which these multiple proteoforms are generated and how they contribute to freezability remain unknown.

Among the proteins associated with poor freezability semen we detected proteins functionally linked with the aldehyde metabolic process and tricarboxylic acid cycle. Moreover we detected proteins assisted in cytoskeletal organization. It has to be strongly underlined that many of these proteins (PASCIN2, PGD, TALDO1, TKT) and/or proteoforms were detected in higher quantities in supernatant following cryopreservation of PF semen. This suggests that there is a relationship between the presence of some proteins enriched in fresh spermatozoa of PF and their leakage after cryopreservation. The exact reason for this phenomenon is unknown. The different protein compositions of spermatozoa of good and poor freezability might arise from genetic differences and/or from differences in spermatogenesis. Also, we speculate that these differences are because of negative changes in properties of the sperm membrane, perhaps arising from previous infection or inflammation of semen, as suggested by elevated levels of acute phase proteins in the seminal plasma of PF [37]. Sub-lethal damage to the sperm membrane might result in more efficient extraction of proteins from fresh spermatozoa of PF and their leakage after cryopreservation.

We also identified proteins released in greater quantities from spermatozoa after cryopreservation of PF semen which were not distinguished in fresh spermatozoa of PF. These proteins included Ca^2+^ binding proteins (EHD1, Calr), Ldhb, nuclear proteins (Ran, EEF1G) and chaperones (HSPA5, Hyou1) involved in metabolism, correct folding of proteins and stress response. The leakage of Ca^2+^- binding proteins and metabolic enzymes from sperm might be part of the mechanism responsible for the decrease in motile sperm in the PF group after cryopreservation. Moreover, as discussed above, the greater leakage of cytosolic enzymes suggests that cryopreservation significantly affects the plasmalemma integrity of PF sperm, leading to an increased leakage of cytoplasmic proteins. The disruption of sperm membrane integrity was confirmed by the presence of plasma membrane (Calr, EHD1, Pascin2) proteins released in higher amount in PF after cryopreservation. String analysis revealed strong interaction between studied proteins especially HSPA5, PDIA3, CALR, GANAB and HYOU1 suggesting its functional link with protein folding and response to stress.

The results of this study complement our recently published analysis of the seminal plasma proteome and its relationship to freezability [37]. Because the seminal plasma and spermatozoa used in these experiments came from the same semen samples comparative analysis of protein changes in both sources is now available. The results of both studies demonstrate that protein component of spermatozoa and the media surrounding them (the seminal plasma) influence carp freezability. Our previous study found that PF is related to an elevated protein level, which reflects infection or inflammation of the reproductive tract, leading to subtle changes in sperm structure. GF is related to higher levels of the proteins involved in the maintenance of sperm membrane integrity and antioxidative protection. This is consistent with the results presented here, which show that sperm with GF show enhanced maintenance of membrane fluidity, antioxidative protection, flagella organization, sperm motility and energy production. It must be emphasized, however, that different proteins are involved in these functions in seminal plasma and spermatozoa. For example Wap65 seems to be associated with antioxidative protection in seminal plasma while glutathione S-transferase (GST), 4-rimethylaminobutyraldehyde dehydrogenase (ALDH9A1) and glutaredoxin in spermatozoa. In summary, it appears that proteins in seminal plasma and spermatozoa influence the freezability of carp semen via different mechanisms. At present it is unknown which of these potential sources of bioindicators offers the better prediction of sperm resilience to long-term cryopreservation.

Taken together, our data demonstrate that variability in cryoresistance can be attributed to differences in protein composition of spermatozoa. Good freezability is related to higher concentrations of the proteins involved in the maintenance of flagella structure, membrane fluidity, efficient control of Ca^2+^ and sperm motility, energy production and antioxidative protection, which reflect the full maturation status of spermatozoa of GF. In contrast PF seems to be related to incomplete maturation or aging of semen and to the presence of proteins shown to be leaked in higher quantities from cryopreserved spermatozoa of PF semen.

Moreover particular proteoforms of proteins related to proper protein folding, energy supply, and methylation status might be critical for either the positive or negative relationship with cryopreservation success. The obtained results would create a platform for future studies designed to assess the functional significance of specific proteins in cryoresistance. Such studies should be based on multiparameter assessment of sperm characteristics (kinetics, structural, biochemical and functional) after cryopreservation [54, 55] to distinguish carp semen of good and poor freezability and to validate the usefulness of identified sperm proteins as potential biomarkers of freezability. At present it is not known whether the dynamics of proteomic changes in semen that affect its freezability display seasonality. This possibility should be investigated in future studies. The results of such research would improve our understanding of the mechanics of sperm freezability in fish, especially carp.

## Supporting information

S1 Table Sperm motion parameters (measured using CASA system) of individual semen sample of good and poor freezability before and after cryopreservation. (XLSX)

S2 Table Experimental set up for CyDye ^TM^ labeling of six spermatozoa of good freezability semen (GF 1–6) and spermatozoa of poor freezability semen (PF 1–6) (1–6) with the incorporation of a pooled internal standard. (DOCX)

S3 Table Experimental set up for CyDye ^TM^ labeling of six supernatant after cryopreservation of good semen (supernatant of GF 1–6) and six supernatant after cryopreservation of poor freezability semen (supernatant of PF 1–6) with the incorporation of a pooled internal standard. (DOCX)

## References

[1] Labbe C, Martoriati A, Devaux A, Maisse G. Effect of sperm cryopreservation on sperm DNA stability and progeny development in rainbow trout. Molecular Reproduction and Development 2001;60:397–404. 11599051 10.1002/mrd.1102

[2] Zilli L, Schiavone R, Zonno V, Storelli C, Vilella S. Evaluation of DNA damage in Dicentrarchus labrax sperm following cryopreservation. Cryobiology 2003;47:227–235. 14697734 10.1016/j.cryobiol.2003.10.002

[3] Zilli L, Schiavone R, Zonno V, Rossano R, Storelli C, Vilella S. Effect of cryopreservation on sea bass sperm proteins. Biology of Reproduction 2005;72:1262–1267. doi: 10.1095/biolreprod.104.036202 15659707 10.1095/biolreprod.104.036202

[4] Martínez-Páramo S, Pérez-Cerezales S, Gómez-Romano F, Blanco G, Sánchez JA, Herráez MP. Cryobanking as tool for conservation of biodiversity: effect of brown trout sperm cryopreservation on the male genetic potential. Theriogenology 2009;71:594–604. doi: 10.1016/j.theriogenology.2008.09.034 18976804 10.1016/j.theriogenology.2008.09.034

[5] Martínez-Páramo S, Diogo P, Dinis MT, Herráez MP, Sarasquete C, Cabrita E. Sea bass sperm freezability is influenced by motility variables and membrane lipid composition but not by membrane integrity and lipid peroxidation. Animal Reproduction Science 2012;131:211–218. doi: 10.1016/j.anireprosci.2012.03.008 22503480 10.1016/j.anireprosci.2012.03.008

[6] Cabrita E, Sarasquete C, Martınez-Paramo S, Robles V, Beirao J, Perez-Cerezales S, et al Cryopreservation of fish sperm: applications and perspectives. Applied Ichthyology 2010;26:623–635.

[7] Yoon SJ, Rahman MS, Kwon WS, Ryu DY, Park YJ, Pang MG. Proteomic identification of cryostress in epididymal spermatozoa. J Anim Sci Biotechnol. 2016 11 21;7:67 doi: 10.1186/s40104-016-0128-2 27895910 10.1186/s40104-016-0128-2 PMC5117493

[8] Gwo JC, Kurokura H, Hirano R. Cryopreservation of spermatozoa from rainbow trout, common carp and marine puffer. Bulletin of Japan Social Science Fish 1993;59:777–782.

[9] Linhart O, Rodina M, Cosson J. Cryopreservation of sperm in common carp *Cyprinus carpio*: Sperm motility and hatching success of embryos. Cryobiology 2000;41:241–250. 11161556 10.1006/cryo.2000.2284

[10] Horváth Á, Miskolczi E, Urbányi B. Cryopreservation of common carp sperm. Aquatic Living Resources 2003;16:457–460.

[11] Warnecke D, Pluta HJ. Motility and fertilizing capacity of frozen/thawed common carp (*Cyprinus carpio* L.) sperm using dimethyl-acetamide as the main cryoprotectant. Aquaculture 2003;215:167–185.

[12] Horokhovatskyi Y, Sampels S, Cosson J, Linhart O, Rodina M, Fedorov P, et al Lipid composition in common carp (*Cyprinus carpio*) sperm possessing different cryoresistance. Cryobiology 2016;73:282–5. doi: 10.1016/j.cryobiol.2016.08.005 27574978 10.1016/j.cryobiol.2016.08.005

[13] Babiak I, Glogowski J, Brzuska E, Szumiec J, Adamek J. Cryopreservation of sperm of common carp, *Cyprinus carpio* L. Aquaculture Research 1997;28:567–571.

[14] Rurangwa E, Kime DE, Ollevier F, Nash JP. The measurements of sperm motility and factors affecting sperm quality in cultured fish. Aquaculture 2004;234:1–28.

[15] Jobim MIM, Oberst ER, Salbego CG, Souza DO, Wald VB, Tramontina F, et al Two-dimensional polyacrylamide gel electrophoresis of bovine seminal plasma proteins and their relation with semen freezability. Theriogenology 2004;61:255–266. 14662126 10.1016/s0093-691x(03)00230-9

[16] Jobim MI, Trein C, Zirkler H, Gregory RM, Sieme H, Mattos RC. Two-dimensional polyacrylamide gel electrophoresis of equine seminal plasma proteins and their relation with semen freezability. Theriogenology 2011;76:765–71. doi: 10.1016/j.theriogenology.2011.04.010 21601917 10.1016/j.theriogenology.2011.04.010

[17] Rickard JP, Leahy T, Soleilhavoup C, Tsikis G, Labas V, Harichaux G, Lynch GW, Druart X, de Graaf SP. The identification of proteomic markers of sperm freezing resilience in ram seminal plasma. Journal of Proteomics 2015;126:303–311. doi: 10.1016/j.jprot.2015.05.017 26025878 10.1016/j.jprot.2015.05.017

[18] Rickard JP, Schmidt RE, Maddison JW, Bathgate R, Lynch GW, Druart X, de Graaf SP. Variation in seminal plasma alters the ability of ram spermatozoa to survive cryopreservation. Reproduction, Fertility and Development 2016;28:516–523. 10.1071/RD14123 25138237

[19] Soleilhavoup C, Tsikis G, Labas V, Harichaux G, Kohnke PL, Dacheux JL, Guérin Y, Gatti JL, de Graaf SP, Druart X. Ram seminal plasma proteome and its impact on liquid preservation of spermatozoa. Journal of Proteomics 2014;109:245–260. doi: 10.1016/j.jprot.2014.07.007 25053255 10.1016/j.jprot.2014.07.007

[20] Yeste M. Sperm cryopreservation update: Cryodamage, markers, and factors affecting the sperm freezability in pigs. Theriogenology. 2016;85:47–64. doi: 10.1016/j.theriogenology.2015.09.047 26506124 10.1016/j.theriogenology.2015.09.047

[21] Moura AA, Memili E. Functional aspects of seminal plasma and sperm proteins and their potential as molecular markers of fertility. Animal Reproduction. 2016;13:191–199.

[22] Casas I, Sancho S, Ballester J, Briz M, Pinart E, Bussalleu E, et al The HSP90AA1 sperm content and the prediction of the boar ejaculate freezability. Theriogenology 2010;74:940–950. doi: 10.1016/j.theriogenology.2010.04.021 20580074 10.1016/j.theriogenology.2010.04.021

[23] Vilagran I, Castillo J, Bonet S, Sancho S, Yeste M, Estanyol JM, et al Acrosin-binding protein (ACRBP) and triosephosphate isomerase (TPI) are good markers to predict boar sperm freezing capacity. Theriogenology 2013;80:443–450. doi: 10.1016/j.theriogenology.2013.05.006 23768753 10.1016/j.theriogenology.2013.05.006

[24] Vilagran I, Yeste M, Sancho S, Casas I, del Álamo MMR, Bonet S. Relationship of sperm small heat-shock protein 10 and voltage-dependent anion channel 2 with semen freezability in boars. Theriogenology 2014;82:418–426. doi: 10.1016/j.theriogenology.2014.04.023 24933094 10.1016/j.theriogenology.2014.04.023

[25] Wang P, Wang YF, Wang H, Wang CW, Zan LS, Hu JH, Li QW, Jia YH, Guo-Ji Ma GJ. HSP90 expression correlation with the freezing resistance of bull sperm. Zygote. 2014;22:239–245. doi: 10.1017/S096719941300004X 23506739 10.1017/S096719941300004X

[26] Valencia J, Gómez G, López W, Mesa H, Henao FJ. Relationship between HSP90a, NPC2 and L-PGDS proteins to boar semen freezability. J Animal Science and Biotechnology 2017;8:21 10.1186/s40104-017-0151-y PMC5335742 28270911

[27] Jiang XP, Wang SQ, Wang W, Xu Y, Xu Z, Tang JY, et al Enolase1 (ENO1) and glucose-6-phosphate isomerase (GPI) are good markers to predict human sperm freezability. Cryobiology. 2015;71:141–145. doi: 10.1016/j.cryobiol.2015.04.006 25910678 10.1016/j.cryobiol.2015.04.006

[28] Rego JP, Martins JM, Wolf CA, van Tilburg M, Moreno F, Monteiro-Moreira AC, et al Proteomic analysis of seminal plasma and sperm cells and their associations with semen freezability in Guzerat bulls. J Anim Sci. 2016;94:5308–5320. doi: 10.2527/jas.2016-0811 28046165 10.2527/jas.2016-0811

[29] Chen X, Zhu H, Hu C, Hao H, Zhang J, Li K, Zhao X, Qin T, Zhao K, Zhu H, Wang D. Identification of differentially expressed proteins in fresh and frozen–thawed boar spermatozoa by iTRAQ-coupled 2D LC–MS/MS. Reproduction. 2014;147:321–330. doi: 10.1530/REP-13-0313 24357664 10.1530/REP-13-0313

[30] Bogle OA, Kumar K, Attardo-Parrinello C, Lewis SEM, Estanyol JM, Ballesc JL, Oliva R. Identification of protein changes in human spermatozoa throughout the cryopreservation process. Andrology. 2017;5:10–22. doi: 10.1111/andr.12279 27860400 10.1111/andr.12279

[31] Yoon SJ, Rahman MS, Kwon WS, Ryu DY, Park YJ, Pang MG. Proteomic identification of cryostress in epididymal spermatozoa. J Anim Sci Biotechnol. 2016;21;7:67 doi: 10.1186/s40104-016-0128-2 27895910 10.1186/s40104-016-0128-2 PMC5117493

[32] Rickard JP, Schmidt RE, Maddison JW, Bathgate R, Lynch GW, Druart X, de Graaf SP. Variation in seminal plasma alters the ability of ram spermatozoa to survive cryopreservation. Reproduction, Fertility and Development 2014;28:516–23. 10.1071/RD14123 25138237

[33] Kime DE, Van Look KJ, McAllister BG, Huyskens G, Rurangwa E, Ollevier F. Computer-assisted sperm analysis (CASA) as a tool for monitoring sperm quality in fish. Comp Biochem Physiol C Toxicol Pharmacol. 2001;130:425–33. 11738630 10.1016/s1532-0456(01)00270-8

[34] Lahnsteiner F. Semen cryopreservation in Salmonidae and in Northern pike. Aquaculture Research. 2000;31:245–258.

[35] Rurangwa E, Volckaert F, Huyskens G, Kime DE, Ollevier F. Quality control of refrigerated and cryopreserved semen using Computer-Assisted Sperm Analysis (CASA), viable staining and standardized fertilization in African catfish (*Clarias gariepinus*). Theriogenology. 2001;55:751–69. 11245263 10.1016/s0093-691x(01)00441-1

[36] Dietrich GJ, Dietrich M, Kowalski RK, Dobosz S, Karol H, Demianowicz W, Glogowski J. Exposure of rainbow trout milt to mercury and cadmium alters sperm motility parameters and reproductive success. Aquat Toxicol. 2010;97:277–84. doi: 10.1016/j.aquatox.2009.12.010 20044150 10.1016/j.aquatox.2009.12.010

[37] Dietrich MA, Irnazarow I, Ciereszko A. Proteomic identification of seminal plasma proteins related to the freezability of carp semen. Journal of Proteomics 2017 doi: 10.1016/j.jprot.2017.04.015 10.1016/j.jprot.2017.04.015 28450256

[38] Kopeika EF. Instruction on low temperature preservation of sperm carp. All-Union Scientific and Productive Society on Fish breeding, Moscow 1986. p 11 (in Russian).

[39] Dietrich MA, Dietrich GJ, Mostek A, Ciereszko A. Motility of carp spermatozoa is associated with profound changes in the sperm proteome. Journal of Proteomics 2016;138:124–135. doi: 10.1016/j.jprot.2016.02.029 26926441 10.1016/j.jprot.2016.02.029

[40] Dietrich MA, Nynca J, Bilinska B, Kuba J, Kotula-Balak M, Karol H, et al Identification of parvalbumin-like protein as a major protein of common carp (*Cyprinus carpio* L) spermatozoa which appears during final stage of spermatogenesis. Comparative Biochemistry and Physiology B-Biochemistry & Molecular Biology. 2010;157:220–227. 10.1016/j.cbpb.2010.06.007 20601062

[41] Meccariello R., Chianese R, Ciaramella V, Fasano S, Pierantoni R. Molecular chaperones, cochaperones, and ubiquitination/deubiquitination system: involvement in the production of high quality spermatozoa. Biomed Res Int. 2014;561426 doi: 10.1155/2014/561426 25045686 10.1155/2014/561426 PMC4089148

[42] Tipler CP, Hutchon SP, Hendil K, Tanaka K, Fishel S, Mayer RJ. Purification and characterization of 26S proteasomes from human and mouse spermatozoa. Molecular Human Reproduction 1997;3:1053–1060. 9464850 10.1093/molehr/3.12.1053

[43] Sutovsky P. Sperm proteasome and fertilization. Reproduction. 2011;142:1–14. doi: 10.1530/REP-11-0041 21606061 10.1530/REP-11-0041

[44] Rickard JP, Schmidt RE, Maddison JW, Bathgate R, Lynch GW, Druart X, et al Variation in seminal plasma alters the ability of ram spermatozoa to survive cryopreservation. Reproduction, Fertility and Development 2016;28:516–523. 10.1071/RD14123 25138237

[45] de Mateo S, Castillo J, Estanyol JM, Ballesca JL, Oliva R. Proteomic characterization of the human sperm nucleus. Proteomics 2011;11:2714–2726. doi: 10.1002/pmic.201000799 21630459 10.1002/pmic.201000799

[46] Sampson MJ, Decker WK, Beaudet AL, Ruitenbeek W, Armstrong D, Hicks MJ, et al Immotile sperm and infertility in mice lacking mitochondrial voltage-dependent anion channel type 3. J. Biol. Chem. 2001;276:39206–39212. 11507092 10.1074/jbc.M104724200

[47] Shoshan-Barmatz V, De Pinto V, Zweckstetter M, Raviv Z, Keinan N, Arbel N. VDAC, a multi-functional mitochondrial protein regulating cell life and death. Mol. Aspects Med. 2010;31:227–285. doi: 10.1016/j.mam.2010.03.002 20346371 10.1016/j.mam.2010.03.002

[48] Hinsch K-D, De Pinto V, Aires VA, Schneider X, Messina A, Hinsch E. Voltage-dependent anion-selective channels VDAC2 and VDAC3 are abundant proteins in bovine outer dense fibers, a cytoskeletal component of the sperm flagellum. J. Biol. Chem. 2004;279:15281–15288. doi: 10.1074/jbc.M313433200 14739283 10.1074/jbc.M313433200

[49] Liu B, Tang M, Han Z, Li J, Zhang J, Lu P, et al Co-incubation of human spermatozoa with anti-VDAC antibody reduced sperm motility. Cell Physiol Biochem. 2014;33:142–50. doi: 10.1159/000356657 24481077 10.1159/000356657

[50] Kwon WS, Park YJ, Mohamed el-SA, Pang MG. Voltage-dependent anion channels are a key factor of male fertility. Fertil Steril. 2013;99:354–61. doi: 10.1016/j.fertnstert.2012.09.021 23062735 10.1016/j.fertnstert.2012.09.021

[51] Li P, Hulak M, Koubek P, Sulc M, Dzyuba B, Boryshpolets S, et al Ice-age endurance: the effects of cryopreservation on proteins of sperm of common carp, *Cyprinus carpio* L. Theriogenology 2010;74:413–423. doi: 10.1016/j.theriogenology.2010.02.024 20570330 10.1016/j.theriogenology.2010.02.024

[52] Krasznai Z, Morisawa M, Morisawa S, Krasznai ZT, Tron L, Gaspar R, et al Role of ion channels and membrane potential in the initiation of carp sperm motility. Aquatic Living Resources. 2003;16:445–449.

[53] Ju TK, Huang FL. MSAP, the meichroacidin homolog of carp (*Cyprinus carpio*), differs from the rodent counterpart in germline expression and involves flagellar differentiation. Biol Reprod. 2004;71:1419–29. doi: 10.1095/biolreprod.104.030346 15215198 10.1095/biolreprod.104.030346

[54] Casas I, Sancho S, Briz M, Pinart E, Bussalleu E, Yeste M, et al Freezability prediction of boar ejaculates assessed by functional sperm parameters and sperm proteins. Theriogenology 2009;72:930–948. doi: 10.1016/j.theriogenology.2009.07.001 19651432 10.1016/j.theriogenology.2009.07.001

[55] Yoon SJ, Kwon WS, Rahman MS, Lee JS, Pang MG. A novel approach to identifying physical markers of cryo-damage in bull spermatozoa. PLoS One. 2015 4;10(5):e0126232 doi: 10.1371/journal.pone.0126232 25938413 10.1371/journal.pone.0126232 PMC4418755

[56] Vizcaíno JA, Csordas A, del-Toro N, Dianes JA, Griss J, Lavidas I, Mayer G, Perez-Riverol Y, Reisinger F, Ternent T, Xu QW, Wang R, Hermjakob H (2016). 2016 update of the PRIDE database and related tools. Nucleic Acids Res 44(D1): D447–D456. doi: 10.1093/nar/gkv1145 26527722 10.1093/nar/gkv1145 PMC4702828

