# Supplementary material for: Proteomic characterization of fresh spermatozoa and supernatant after cryopreservation in relation to freezability of carp (Cyprinus carpio L) semen
Source: PLoS One. 2018 Mar 22;13(3):e0192972. doi: 10.1371/journal.pone.0192972 (PMC5863941; doi:10.1371/journal.pone.0192972)
Supplement: S3 Table — (DOCX) [file pone.0192972.s003.docx]

**S3 Table** Experimental set up for CyDye ^TM^ labeling of six supernatant after cryopreservation of good semen (supernatant of GF 1-6) and six supernatant after cryopreservation of poor freezability semen (supernatant of PF 1-6) with the incorporation of a pooled internal standard.

|  | Cy2 | Cy3 | Cy5 |
| --- | --- | --- | --- |
| Gel 1 | 50 µg Pooled Std. | 50 µg supernatant of GF 1 | 50 µg supernatant of PF 1 |
| Gel 2 | 50 µg Pooled Std. | 50 µg supernatant of GF 2 | 50 µg supernatant of PF 2 |
| Gel 3 | 50 µg Pooled Std. | 50 µg supernatant of GF 3 | 50 µg supernatant of PF 3 |
| Gel 4 | 50 µg Pooled Std. | 50 µg supernatant of PF 4 | 50 µg supernatant of GF 4 |
| Gel 3 | 50 µg Pooled Std. | 50 µg supernatant of PF 5 | 50 µg supernatant of GF 5 |
| Gel 4 | 50 µg Pooled Std. | 50 µg supernatant of PF 6 | 50 µg supernatant of GF 6 |
| Total Internal standard 300 µg (from a pool of 12 x 25 µg, each from supernatant of GF (sample 1 to 6) and supernatant of GF (sample 1 to 6). | | | |
